# The Annual Meeting of the Southern Surgical and Gynecological Association

**Published:** 1888-09

**Authors:** 


THE ANNUAL MEETING OF THE SOUTHERN SURGICAL AND GYNECOLOGICAL ASSOCIATION to be held at Birmingham, Alabama, on September 11, 12 and 13, 1888, promises to be a meeting of much interest. W. D. Haggard, M. D., of Nashville, will, as President, make the Annual Address, and W. F. Hyer, of Holly Springs, Miss., will make the Annual Oration. Numerous interesting papers are to be read and discussed on the occasion by prominent men in the profession.

"The Association will convene in the hall of the Young Men's Christian Association, at 10 o'clock a.m. each day. The Annual Oration will be delivered at O'Brien's Opera House on the evening of the first day's session, at which time the Mendelssohn Club of Birmingham will give a concert for the entertainment of the Association. Entertainments have been arranged by the local committee to take up all the hours not occupied by the sessions. Hotels and railroads will give reduced rates, but only those holding certificates, signed by the ticket agent at point where through ticket to place of meeting was purchased, will be entitled to the two-thirds reduction in return fare."

